# HPV DNA Testing and Mobile Colposcopy for Cervical Precancer Screening in HIV Positive Women: A Comparison Between Two Settings in Ghana and Recommendation for Screening

**DOI:** 10.1177/10732748241244678

**Published:** 2024-04-02

**Authors:** Kofi Effah, Richard Anthony, Ethel Tekpor, Joseph E. Amuah, Comfort M. Wormenor, Georgina Tay, Smith E. Y. Kraa, Angela M. Katso, Christiana A. Akonnor, Seyram Kemawor, Stephen Danyo, Bernard H. Atuguba, Nana Owusu M. Essel, Patrick K. Akakpo

**Affiliations:** 1 Catholic Hospital, Battor, Volta Region, Ghana; 2 Tema General Hospital, Tema, Ghana; 3 School of Epidemiology and Public Health, Faculty of Medicine, University of Ottawa, Ottawa, ON, Canada; 4 Department of Emergency Medicine, College of Health Sciences, 12357 Faculty of Medicine and Dentistry, University of Alberta, Edmonton, AB, Canada; 5 Department of Pathology, School of Medical Sciences, Clinical Teaching Center, 107841 University of Cape Coast, Cape Coast, Ghana

**Keywords:** HIV, rural-urban disparities, human papillomavirus infection, cervical precancer screening, mobile colposcopy, human papillomavirus DNA testing

## Abstract

**Introduction:**

Women living with HIV (WLHIV) have higher prevalence and persistence rates of high-risk human papillomavirus (hr-HPV) infection with a six-fold increased risk of cervical cancer. Thus, more frequent screening is recommended for WLHIV.

**Objectives:**

This retrospective descriptive cross-sectional study was conducted to investigate and compare the prevalence of hr-HPV infection and abnormal findings on mobile colposcopy in two cohorts of WLHIV following cervical screening in rural and urban settings in Ghana.

**Methods:**

Through the mPharma 10 000 Women Initiative, WLHIV were screened via concurrent hr-HPV DNA testing (MA-6000; Sansure Biotech Inc., Hunan, China) and visual inspection (Enhanced Visual Assessment [EVA] mobile colposcope; MobileODT, Tel Aviv, Israel) by trained nurses. The women were screened while undergoing routine outpatient reviews at HIV clinics held at the Catholic Hospital, Battor (rural setting) and Tema General Hospital (urban setting), both in Ghana.

**Results:**

Two-hundred and fifty-eight WLHIV were included in the analysis (rural, n = 132; urban, n = 126). The two groups were comparable in terms of age, time since HIV diagnosis, and duration of treatment for HIV. The hr-HPV prevalence rates were 53.7% (95% CI, 45.3–62.3) and 48.4% (95% CI, 39.7–57.1) among WLHIV screened in the rural vs urban settings (*p*-value = .388). Abnormal colposcopy findings were found in 8.5% (95% CI, 5.1–11.9) of the WLHIV, with no significant difference in detection rates between the two settings (*p*-value = .221). Three (13.6%) of 22 women who showed abnormal colposcopic findings underwent loop electrosurgical excision procedure (LEEP), leaving 19/22 women from both rural and urban areas with pending treatment/follow-up results, which demonstrates the difficulty faced in reaching early diagnosis and treatment, regardless of their area of residence. Histopathology following LEEP revealed CIN III in 2 WLHIV (urban setting, both hr-HPV negative) and CIN I in 1 woman in the rural setting (hr-HPV positive).

**Conclusions:**

There is a high prevalence of hr-HPV among WLHIV in both rural and urban settings in this study in Ghana. Concurrent HPV DNA testing with a visual inspection method (colposcopy/VIA) reduces loss to follow-up compared to performing HPV DNA testing as a standalone test and recalling hr-HPV positive women for follow up with a visual inspection method. Concurrent HPV DNA testing and a visual inspection method may also pick up precancerous cervical lesions that are hr-HPV negative and may be missed if HPV DNA testing is performed alone.

## Introduction

Women living with HIV (WLHIV) are at a higher risk of infection with high-risk human papillomavirus (hr-HPV) compared to the general population and are more likely to experience mixed-type HPV infections, persistent hr-HPV infection, and progression to cervical dysplasia.^1^ Recent estimates point to a total of 342 307 people living with HIV in Ghana (prevalence, 1.6%), with a higher prevalence among women (2.5%).^2,3^ Just like other women living in low- and middle-income countries (LMICs), WLHIV may miss opportunities for cervical precancer screening at enrolment and during visits to HIV clinics for reviews, viral load monitoring, and highly-active antiretroviral therapy (HAART) refills. Such missed opportunities increase the likelihood that WLHIV will present with advanced cervical cancer with a poor prognosis. Integrating cervical screening into routine care for WLHIV has been recommended as an effective approach to improve overall screening uptake and attain early precancer detection and management among WLHIV.^4^ Ghana currently has no policy in place for cervical precancer screening among women in the general population and is in the process of rolling out a cervical precancer screening project in the form of hr-HPV DNA screening for WLHIV.^5^ To successfully implement this program, formative research pertaining to the epidemiologic characteristics of WLHIV in various settings is pertinent for a number of reasons. In developing countries like Ghana, rural and urban regions tend to show differences in social and physical environments that result in different disease burdens and health outcomes. In addition to the overall poorer health infrastructure in Ghana, rural inhabitants typically experience inequity in the form of poorer access to health services compared to urban dwellers.^6^

Among WLHIV in Ghana, research pertaining to cervical precancer screening has largely focused on predictors of the intention to undergo screening,^7,8^ knowledge of HPV and experiences of screening,^9^ perceptions of their susceptibility to cervical cancer,^10^ barriers to and facilitators of screening,^11^ as well as the distribution of hr-HPV genotypes and associated factors.^12^ The findings of these studies might be instrumental in guiding the pilot and implementation phases of integration programs targeting WLHIV. However, population-based disparities in cervical precancer risk between WLHIV dwelling in rural and urban areas in Ghana are poorly understood. To broaden our understanding of the patterns of rural-urban disparities in cervical cancer risk among WLHIV, we aimed to investigate and compare the prevalence of hr-HPV infection and abnormal colposcopy findings among WLHIV attending HIV clinics in urban and rural areas in Ghana.

## Materials and Methods

### Study Design

This retrospective descriptive cross-sectional study was conducted to investigate and compare the prevalence of hr-HPV infection and abnormal colposcopy findings among WLHIV who underwent cervical screening using concurrent hr-HPV DNA testing (MA-6000; Sansure Biotech Inc., Hunan, China) and visual inspection (with the Enhanced Visual Assessment [EVA] mobile colposcope; MobileODT, Tel Aviv, Israel). The screening exercises were conducted as part of the mPharma 10 000 Women campaign that sought to provide cervical screening to 10 000 women in Ghana and Nigeria via HPV DNA testing.^13^ The findings of this study constitute a partial report from this campaign, which provided screening to several groups of women in Ghana (total, approximately 6000), including female migrant head porters (*kayayei*), women with sickle cell disease,^14^ women with diabetes, and women living in the remote Nzulezo Stilt Village.^15^ The Ghanaian arm of the initiative came to an end in October 2022. The reporting of this study conforms to STROBE guidelines.^16^

### Study Settings and Participants

The present study involved the screening of 263 consecutive WLHIV aged >20 years who volunteered to undergo registration and cervical precancer screening between February and July 2022. These women were screened while undergoing routine outpatient HAART and clinical monitoring at HIV clinics situated in two secondary hospitals in Ghana (Catholic Hospital, Battor and Tema General Hospital, Tema). Battor is a rural community located at 6°4′ North, 0°25′ East and the administrative capital of the North Tongu District of Ghana. The Catholic Hospital, Battor provides major primary health care services in the district and is supported by local Community-based Health Planning and Services compounds. Tema General Hospital is situated in the Tema Metropolis of Ghana, located at 05°40′ North 00°00′ West, an urban city about 16 miles east of the country’s capital, Accra. It is the premium hospital in the Tema Municipality and an HIV-designated center. The relative locations of the 2 study facilities in which cervical screening was performed are shown in Supplemental Figure 1.

### Study Procedures

Before screening, all WLHIV were given details pertaining to screening procedures, how they would be performed, and any associated benefits and risks. After obtaining verbal consent, a screening nurse collected data on social, demographic, and medical characteristics using a structured questionnaire in routine use at the Cervical Cancer Prevention and Training Centre (CCPTC), Catholic Hospital, Battor. Thereafter, screening was performed by way of cervical sampling for laboratory HPV DNA testing and EVA mobile colposcopy in the same setting. All cervical samples collected at both study sites were processed and tested at the central laboratory of the CCPTC using the MA-6000 hr-HPV DNA platform within 1 week of collection. Data regarding the results of HPV DNA testing and sociodemographic and clinical details collected using the questionnaire were entered into REDCap 11.0.3 (Vanderbilt University, Nashville, TN, USA) and stored in secure research databases hosted and managed by the CCPTC. Prior to the data analysis, the databases were queried, and data extracted and anonymized. All patient details have also been de-identified in this report. The study outcome(s) of interest was either a positive hr-HPV DNA test (hr-HPV positivity) and/or an abnormal (‘positive’) finding on EVA mobile colposcopy (hereafter EVA ‘positivity’).

### Ethical Considerations

The study complied with the Declaration of Helsinki (1964) and its later amendments. All study participants provided verbal informed consent before questionnaire administration, cervical sample collection, and visual inspection procedures. The consent procedure was approved by the Ethical Review Committee of the Catholic Hospital, Battor (approval no. CHB-ERC 0120/06/22), which also gave the researchers permission to publish the study findings retrospectively.

### Sample Size

No sample size determination was performed before study initiation because the WLHIV were screened in the context of service provision, not as part of a research study. Also, we did not find any population-based studies assessing WLHIV in rural and urban settings in Ghana that would serve as an objective basis for sample size calculation. Convenience sampling was thus used, including WLHIV who attended HIV clinics between February and July 2022 in the rural setting and April 2022 for the outreach in the urban setting.

### Cervical HPV Specimen Collection and EVA Colposcopy

At both study sites, cervical specimens were taken and mobile colposcopy was performed by trained and experienced nurses from the CCPTC.^17^ The procedures were performed with each woman lying in the dorsal lithotomy position. A sterile vaginal speculum was gently placed to achieve cervical exposure and a Bioline sterile swab stick (Bioline Diagnostics LLP, New Delhi, India) was used to take cervical samples in circular motions. The collected specimen was placed in a plain specimen collection tube, capped, and sent to the laboratory for processing and testing.

Immediately after specimen collection, colposcopy was performed using the EVA system 3.0 to visualize the presence of cervical abnormalities. Specifically, the hardware used comprised a Samsung J500 phone, a white LED light source, handle, protective case, and a lens that enabled images to be taken at a working distance in addition to magnification. Colposcopic findings were reported using the 2011 International Federation for Cervical Pathology and Colposcopy (IFCPC) terminology (adequacy, transformation zone type, lesion seen, no lesion seen, etc).^18^ The findings thereof were interpreted as ‘positive’ if any clinically-relevant abnormal finding or leukoplakia (hyperkeratosis) was found or ‘negative’ otherwise.

### Laboratory Testing: MA-6000 HPV DNA Extraction and Assay

Cervical samples collected from the WLHIV before visual inspection were processed and tested for hr-HPV DNA using the MA-6000 platform strictly according to the instructions of the manufacturer.^19,20^ In brief, a pure DNA fraction was isolated in solution by adding 5 µl of the manufacturer’s sample release reagent (Sansure Biotech Inc.; catalog no. S3027E) to 5 µl of the cell suspension, mixing, and incubating at room temperature. Then, 45 PCR cycles were run in series, DNA amplification was performed, and fluorescence data were collected. The tests were run using the semi-quantitative module of the MA-6000 platform, which is configured to recognize fifteen HPV genotypes based on these 4 dyes: HEX, for the detection of human beta-globin as an internal control; ROX, for the detection of HPV 31, 33, 35, 39, 45, 51, 52, 53, 56, 58, 59, 66, and 68 without distinction as *other* hr-HPV DNA; CY5 for the detection of HPV 16; and FAM, for the detection of HPV18. The test outputs were read and interpreted in strict accordance with the guidelines of the manufacturer.

### Treatment and Follow-Up Strategy

In line with algorithms in place at the CCPTC, hr-HPV negative WLHIV with normal colposcopic findings were counseled to undergo rescreening in 3 years. For hr-HPV negative WLHIV with abnormal colposcopic findings (major changes), the ideal approach would have been a biopsy of the lesion to guide management. Because women generally had to pay out of pocket and had limited funds, many on counseling preferred a ‘diagnostic loop electrosurgical excision procedure (LEEP)’ which is both diagnostic and therapeutic and reduces the cost associated with multiple visits and having to pay for histopathology twice. This was done for the two women who had CIN III. Women with minor changes (likely to be CIN I) were given the option of conservative management (rescreening with a visual inspection method in 6 months/1 year) as the lesions may clear spontaneously. The mPharma 10 000 Women Initiative provided funds for treatment with thermal coagulation and LEEP; however, funds were insufficient to cover biopsies for all lesions before treatment.

Women with cervical leukoplakia (a white patch on the epithelium before applying acetic acid) were counseled to undergo excision (not ablative treatment) because leukoplakia could be benign or have dysplasia or invasive disease beneath it. After excision, follow-up would depend on the histopathology report. For instance, LEEP was performed for one woman with leukoplakia and dense acetowhitening. The histopathology finding was CIN III; thus, she was scheduled for follow-up colposcopy at 6 months/1 year. Women who tested hr-HPV positive but had normal colposcopic findings were counseled on HPV DNA testing after 1 year. They had the option to be followed up at other facilities or our center. For the women who could not afford the follow-up screening, having been entered into our database, we would use the training program of the CCPTC (where each trainee pays for ‘free’ screening of 15 women) to screen them.^21^ We shall be visiting the Tema General Hospital at a later date, at which time women ‘lost to follow-up’ (who could not get follow-up screening on their own) have been called to undergo ‘free’ screening/follow-up. In addition, those who had invalid hr-HPV DNA test results will be recalled for repeat sample collection.

### Statistical Analyses

Categorical variables (e.g., level of education, income status, marital status, hr-HPV positivity, and abnormal colposcopic findings) are summarized using frequencies and proportions. Continuous variables with symmetric distributions are presented as means and standard deviations (SDs), whereas skewed variables are summarized using medians and interquartile ranges (IQRs). The prevalence of hr-HPV infection and abnormal colposcopy findings are reported in percentage form with 95% confidence intervals (CIs). The chi-squared test of independence was used to assess associations between categorical variables and prevalence ratios (PRs) with their 95% CIs were computed to compare positivity rates between the two groups. The independent samples *t*-test was used to compare continuous variables with symmetric distributions between the two groups and the Wilcoxon rank-sum test was used to compare the medians of skewed continuous variables. Finally, odds ratios (ORs) and adjusted ORs were used to compare screening outcomes (hr-HPV infection and abnormal colposcopy findings) between women screened in the two settings with the urban group as the reference. The adjusted analyses were performed to control for all variables included in the study that presented significant differences between the 2 settings. All statistical analyses were performed using Stata version 14.2 (StataCorp LLC, College Station, TX, USA). All hypothesis tests were two-sided and performed at an alpha level of 5%.

## Results

### Recruitment and Selection of WLHIV for Cervical Precancer Screening

**Figure 1.**
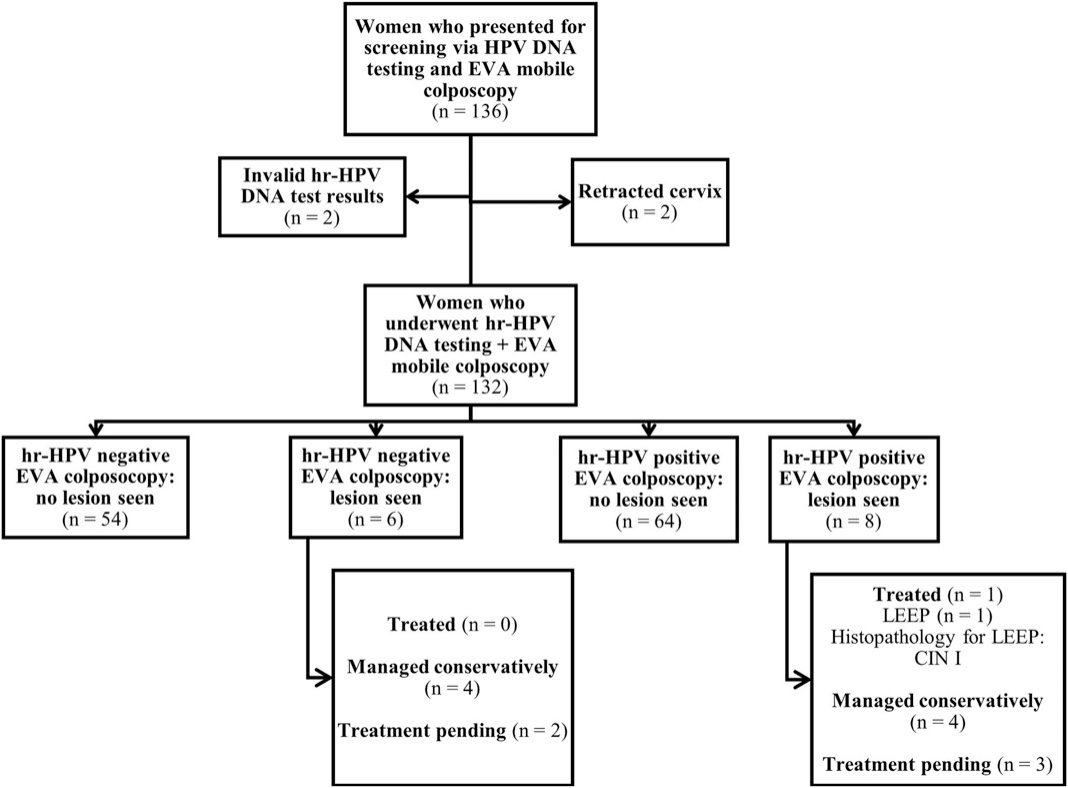
Flow chart for cervical screening in a rural setting using hr-HPV DNA testing and EVA mobile colposcopy. *Abbreviations*: hr-HPV, high-risk human papillomavirus; LEEP, loop electrosurgical excision procedure; CIN, cervical intraepithelial neoplasia; EVA, Enhanced Visual Assessment.

**Figure 2.**
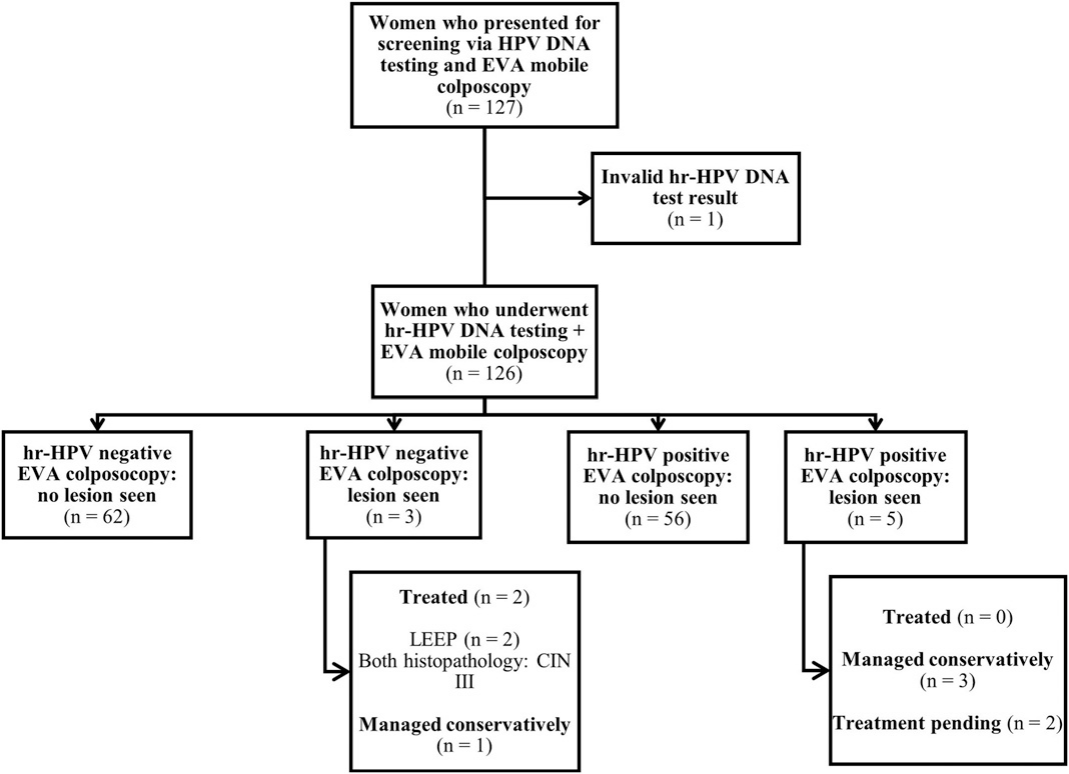
Flow chart for cervical screening in an urban setting using hr-HPV DNA testing and EVA mobile colposcopy. *Abbreviations*: hr-HPV, high-risk human papillomavirus; LEEP, loop electrosurgical excision procedure; CIN, cervical intraepithelial neoplasia; EVA, Enhanced Visual Assessment.

A total of 263 WLHIV receiving care at the HIV clinics of the Catholic Hospital Battor (rural, n = 136) and Tema General Hospital (urban, n = 127) underwent cervical screening by way of concurrent hr-HPV DNA testing and EVA mobile colposcopy. In the rural setting, two of the initial 136 WLHIV were excluded from the analysis on account of retracted cervix and another two had invalid HPV DNA test results (Figure 1). In the urban setting, 1 patient was excluded on account of an invalid HPV DNA test result (Figure 2). Thus, the analyzed dataset comprised the data of 258 WLHIV (rural, n = 132; urban, n = 126).

### Sociodemographic and Clinical Characteristics of Women Attending HIV Clinics who Underwent Cervical Screening in the Rural vs Urban Settings

The sociodemographic, and clinical details of the study participants are shown in Table 1. Women screened in the 2 settings did not show statistically significant differences in some characteristics of interest, such as age (*p*-value = .948), marital status (*p*-value = .840), smoking status (*p*-value = .165), alcohol consumption, and past- or current contraceptive use (*p*-values = .229 and .611, respectively); the frequencies recorded for these variables were similar between the 2 settings. However, they showed statistically significant differences in parity (*p*-value = .036), education level (*p*-value = .009), and monthly income level (*p*-value <.001). There were also no significant differences in the time since HIV diagnosis and duration of HIV treatment between WLHIV screened in the urban and rural settings (*p*-values = .738 and .726, respectively). None had been vaccinated against hr-HPV infection, while 15.9% had previously undergone cervical screening. However, women screened in the rural setting (n = 38; 28.2%) were approximately 12 times more likely to have undergone previous cervical screening compared to their urban counterparts (n = 3, 2.4%) (*p*-value <.001).

**Table 1.** Sociodemographic and Clinical Characteristics of Women Attending HIV Clinics Who Underwent Cervical Screening in Rural Versus Urban Settings.

Sociodemographic Variables	Rural (n = 132)	Urban (n = 126)	*p*-value
Age, mean (SD)	46.4 (12.7)	46.3 (10.3)	.948
Parity, median (IQR)	3 (1, 4)	3 (2, 5)	.036^a^
Religion, n (%)			
Christian	124 (93.9)	119 (94.4)	.188
Muslim	4 (3.0)	7 (5.6)	
Traditionalist/other	4 (3.0)	0 (.0)	
Marital status, n (%)			
Divorced	13.6 (18)	13.5 (17)	.840
Single	17.4 (23)	13.5 (17)	
Widowed	24.2 (32)	24.6 (31)	
Married/cohabiting	44.7 (59)	48.4 (61)	
Education level, n (%)			
None	38 (28.8)	20 (15.9)	.009^a^
Primary	40 (30.3)	27 (21.4)	
Secondary	46 (34.9)	65 (51.6)	
Tertiary	5 (3.8)	6 (4.8)	
Other	3 (2.3)	8 (6.4)	
Earns an income, n (%)	109 (82.5)	108 (85.7)	.491
Monthly income in GH¢^b^, n (%)			
<100	71 (53.8)	33 (26.2)	<.001^a^
100–250	6 (4.6)	12 (9.5)	
250–500	10 (7.6)	28 (22.2)	
>500	16 (12.1)	27 (21.4)	
Prefer not to say/missing	29 (22.0)	26 (20.6)	
National health insurance scheme	107 (81.1)	97 (77.0)	.421
Ever consumed alcohol, n (%)	0 (.0)	0 (.0)	—
Ever smoked, n (%)	2 (1.5)	0 (.0)	.165
Past contraceptive use, n (%)	51 (38.6)	58 (46.0)	.229
Current contraceptive use, n (%)	7 (5.3)	5 (4.0)	.611
Clinical characteristics			
Time since HIV diagnosis, year(s); n (%)			
<1	9 (6.8)	6 (4.8)	.738
1	14 (10.5)	9 (7.1)	
2	11 (8.3)	9 (7.1)	
3	6 (4.5)	8 (6.4)	
>3	93 (69.9)	94 (74.6)	
Currently receiving HIV treatment, n (%)	131 (98.5)	123 (97.6)	.608
Duration of HIV treatment, year(s); n (%)			
<1	9 (6.8)	6 (4.8)	.726
1	14 (10.6)	9 (7.1)	
2	11 (8.3)	9 (7.1)	
3	6 (4.6)	8 (6.4)	
>3	92 (69.7)	94 (74.6)	
Past medical history			
Hypertension, n (%)	16 (12.1)	16 (12.7)	.888
Diabetes mellitus, n (%)	1 (.8)	3 (2.4)	.291
Asthma, n (%)	1 (.8)	3 (2.4)	.291
Tuberculosis, n (%)	0 (.0)	1 (.8)	.305
Previous HPV vaccination, n (%)	0 (.0)	0 (.0)	-
Previous cervical screening, n (%)	38 (28.2)	3 (2.4)	<.001^a^
Previous cervical treatment, n (%)	2 (1.5)	0 (.0)	.165

*Abbreviations*: HIV, human immunodeficiency virus; SD, standard deviation; IQR, interquartile range.

^a^Statistically significant.

^b^Among 218 women who earned an income.

### Prevalence of Screening Characteristics and Outcomes Stratified by Rurality Among the WLHIV

The prevalence rates of hr-HPV infection and abnormal colposcopy findings are shown in Table 2. A little over half of the 258 HIV patients tested hr-HPV positive (51.2%; 95% CI, 45.1–57.3). The hr-HPV positivity rate was slightly higher in the rural group but without statistical significance (rural, 53.7% vs urban, 48.4%; PR, 1.1 [95% CI, .9–1.4; *p*-value = .388]). Abnormal colposcopy findings were found in 8.5% (95% CI, 5.1–11.9) of the 258 WLHIV, with no statistically significant difference in ‘positivity’ rate between the two settings (rural: 10.6% vs urban: 6.3%; PR, 1.7 [95% CI, .7–3.8; *p*-value = .221]). Among hr-HPV-positive WLHIV, there was no difference in mean age between those who showed abnormal colposcopic findings and those who showed normal colposcopic findings (*p*-value = .615). Similarly, for hr-HPV-negative WLHIV, the presence of abnormal colposcopic findings was independent of age (*p*-value = .739).

**Table 2.** Prevalence of Screening Characteristics Stratified by Rurality Among 258 WLHIV Who Underwent Concurrent hr-HPV DNA Testing and EVA Mobile Colposcopy.

Screening Characteristics	Rural (n = 132)	Urban (n = 126)	PR (95% CI)	*p*-value
Abnormal findings on *gross* inspection				
Vulval examination, n (%)	5 (3.8)	4 (3.2)	1.2 (.3–4.3)	.788
Vaginal examination, n (%)	1 (.8)	2 (1.6)	.5 (.0–5.2)	.534
Cervical examination, n (%)	4 (3.0)	11 (8.7)	.3 (.1–1.1)	.054
Infection with specific hr-HPV type(s)				
HPV16, n (%)	7 (5.3)	3 (2.4)	2.2 (.6–8.4)	.224
HPV18, n (%)	8 (6.1)	10 (7.9)	.8 (.3–1.9)	.554
*Other* hr-HPV genotypes, n (%)	65 (49.2)	57 (45.2)	1.1 (.8–1.4)	.520
Single *vs* mixed hr-HPV infections				
HPV16 only, n (%)	2 (1.5)	1 (.8)	1.9 (.2–20.8)	.589
HPV18 only, n (%)	4 (3.0)	2 (1.6)	1.9 (.4–10.2)	.442
*Other* hr-HPV genotypes only, n (%)	58 (43.9)	47 (37.3)	1.2 (.9–1.6)	.278
HPV16 + HPV18, n (%)	2 (1.5)	0 (.0)	-	.165
HPV16 + *other* hr-HPV type(s), n (%)	5 (3.8)	2 (1.6)	2.4 (.5–12.1)	.277
HPV18 + *other* hr-HPV type(s), n (%)	4 (3.0)	8 (6.4)	.5 (.1–1.5)	.206
hr-HPV infection, n (%; 95% CI)	71 (53.7; 45.3–62.3)	61 (48.4; 39.7–57.1)	1.1 (.9–1.4)	.388
Abnormal colposcopy findings, n (%; 95% CI)	14 (10.6; 5.4–15.9)	8 (6.3; 2.1–10.6)	1.7 (.7–3.8)	.221

*Abbreviations*: CI, confidence interval; hr-HPV, high-risk human papillomavirus; PR, prevalence ratio.

Abnormal colposcopy findings: Leukoplakia (white patch on the cervix before applying acetic acid) or acetowhitening after applying acetic acid.

Although not statistically significant, the prevalence of specific hr-HPV genotypes detected tended to be higher in the rural group compared to the urban group for HPV16 (PR, 2.2; 95% CI, .6–8.4; *p*-value = .224) and *other* hr-HPV genotype(s) (PR, 1.1; 95% CI, .8–1.4; *p*-value = .520), but not HPV18 (PR, .8; 95% CI, .3–1.9; *p*-value = .554). Single-genotype hr-HPV infections tended to be higher in the rural group, but without statistical significance: HPV16 only (*p*-value = .589), HPV18 only (*p*-value = .442), and *other* hr-HPV genotype(s) only (*p*-value = .278). Except for HPV18 + *other* hr-HPV genotype(s) (*p*-value = .206) which tended to be higher in the urban group, mixed genotype hr-HPV infections also tended to be higher in the rural group than the urban group, but without statistical significance: HPV16 + HPV18 (*p*-value = .165) and HPV16 + *other* hr-HPV type(s) (*p*-value = .277) (Table 2).

### Comparison of Screening Outcomes (hr-HPV Infection and Abnormal Colposcopy Findings) Between WLHIV Screened in the Two Settings via Concurrent hr-HPV DNA Testing and EVA Mobile Colposcopy

The crude and adjusted ORs of the screening outcomes among the WLHIV screened in the rural setting (compared to those screened in the urban setting) are shown in Table 3. Compared to WLHIV in the urban group, those screened in the rural setting respectively showed 1.2 (95% CI, .8–2.0) times higher and 1.8 (95% CI, .7–4.3) times higher odds of hr-HPV infection and abnormal colposcopic findings, but each without statistical significance. After controlling for characteristics that showed significant differences between the settings (parity, education level, monthly income, and prior cervical screening), the effect estimate for abnormal colposcopy findings (aOR = 2.1; 95% CI, .8–5.8) was similar to the univariable value, whereas that of hr-HPV infection was reversed (aOR = .9; 95% CI, .5–1.5); these multivariable relationships, however, were not statistically significant.

**Table 3.** Crude and Adjusted Odds ratios^a^ of hr-HPV Infection and Abnormal Colposcopy Findings Among WLHIV Screened by Setting (Rural/Urban) via Concurrent hr-HPV DNA Testing and EVA Mobile Colposcopy.

Screening Outcome	Or (95% CI)	*p*-value	aOR^b^ (95% CI)	*p*-value
hr-HPV infection	1.2 (.8–2.0)	.388	.9 (.5–1.5)	.626
Abnormal colposcopy findings	1.8 (.7–4.3)	.226	2.1 (.8–5.8)	.157

*Abbreviations*: OR, crude odds ratio; aOR, adjusted odds ratio; CI, confidence interval; hr-HPV, high-risk human papillomavirus.

Abnormal colposcopy findings: Leukoplakia (white patch on the cervix before applying acetic acid) or acetowhitening after applying acetic acid.

^a^Reference group for all odds ratios is the urban group.

^b^Adjusted for parity, education level, monthly income, and previous cervical screening.

## Discussion

We have examined and compared the prevalence of hr-HPV infection and abnormal colposcopy findings in WLHIV who underwent cervical precancer screening using concurrent HPV DNA testing and EVA mobile colposcopy in rural and urban settings in Ghana. We found that the urban and rural groups were not significantly different in terms of the time since HIV diagnosis and the duration of HIV treatment; however, WLHIV in the rural group were 12 times more likely to have undergone prior cervical screening than those in the urban group. We also recorded no prior HPV vaccinations in either group and found no significant differences in the overall rates of hr-HPV infection (as well as single vs mixed genotype infections) and abnormal colposcopy findings between the groups. Out of 22 women who showed abnormal colposcopic findings, 3 (13.6%) were treated using LEEP. Histopathology results following LEEP revealed CIN III in 2 WLHIV (all screened in the urban setting, both hr-HPV negative) and CIN I in 1 woman (screened in the rural setting, hr-HPV positive). Of the remaining 19 WLHIV, 7 are pending treatment and the remaining 12 are being managed conservatively. All the women who had conservative management had minor changes (thin acetowhitening) at colposcopy. The funding was not enough to cover biopsies of all lesions so women with minor changes (thin acetowhitening), suggestive of CIN I, were scheduled for follow up in 6 to 12 months. This was to prevent overtreatment. Women with major changes (dense acetowhitening) suggestive of CIN II/III were referred to Catholic Hospital, Battor for treatment (LEEP), the cost of which was covered by the project.

The overall hr-HPV positivity rate observed here (51.2%) and the urban-rural stratified prevalence rates (48.4% and 53.7%, respectively) were more than 2 times higher than that reported by the World Health Organization (WHO) (21.3%) for women in the general population of West Africa.^22,23^ These results were also higher than the prevalence of 42.7% (95% CI, 34.4–48.1)^12^ recently reported among WLHIV with a similar demographic profile as our combined cohort screened with the AmpFire platform at an HIV clinic in Cape Coast, a predominantly urban setting. On the other hand, the overall and setting-specific hr-HPV prevalence rates were lower than that reported among HIV-1 seropositive WLHIV (65.6%), also in Cape Coast, screened using the Anyplex II HPV28 assay.^24^ It is worth noting, however, that in addition to the 13 known carcinogenic (HPV16, 18, 31, 33, 35, 39, 45, 51, 52, 56, 58, 59, and 68) and 2 possible carcinogenic (HPV53 and 66) genotypes^25^ detected by the MA-6000 platform we used, the Anyplex II assay also detects 4 other possible carcinogenic HPV genotypes (HPV26, 69, 73, and 82) as ‘hr-HPV’ types. Overall, the results highlight the need to hasten efforts aimed at integrating the delivery of cervical screening within routine HIV care and to increase the access to and utilization of such services among WLHIV in Ghana.

Apart from the disproportionately high rate of hr-HPV and HIV coinfection, 1 similarity we found between both settings in this study was a zero rate of HPV vaccination among the WLHIV. As HPV vaccination is not covered by public insurance in Ghana, it appears that many WLHIV may be unwilling or unable to pay for it, as highlighted in a recent study conducted in Nigeria.^26^ Thus, the cost of HPV vaccination may be a substantial barrier to its uptake, especially for women with lower incomes, which was the rural group in this study. We also observed that women screened in the rural setting were 12 times more likely to have undergone cervical screening than those screened in the urban setting. This is particularly noteworthy as both groups were not significantly different in terms of hr-HPV positivity rate (and thus the apparent risk of cervical precancer), time since HIV diagnosis, and duration of HIV treatment. This is likely because the CCPTC is situated in the Catholic Hospital, Battor where the HIV clinic in the rural setting is situated and also runs regular outreaches to communities within the North Tongu district. Interestingly, however, women in the rural setting showed higher odds compared to those screened in the urban group for hr-HPV infection and abnormal colposcopy findings, although this association did not reach statistical significance. Although the observed relationship persisted for abnormal colposcopy findings after controlling for parity, education level, monthly income, and prior cervical screening, the rural group showed lower odds of hr-HPV infection in the adjusted analysis. Our finding of a higher prior screening rate in the rural group could in turn have influenced the hr-HPV prevalence observed in either group since early detection may affect the HPV prevalence; for instance, hr-HPV-infected women could have been advised on condom use to avoid repeated infections. Our finding with regard to screening utilization disagrees with a study conducted in Kenya which reported 1.14 times higher odds (aOR = 1.14; 95% CI, 1.01–1.30) of cervical screening utilization among urban-dwelling women compared to rural-dwelling women.^27^ It also indicates the presence of rural-urban disparities in cervical screening uptake in Ghana that need to be addressed via public health promotion targeting the comparatively disadvantaged rural WLHIV, while simultaneously ensuring that efforts are more effectively distributed.

Cervical cancer prevention hinges on the effective management of precancerous lesions. Many studies describing cervical screening integration into existing HIV services elsewhere have recommended the use of visual inspection methods by training existing staff, as well as the treatment of screen-positive WLHIV with minor to large lesions.^28^ WLHIV are also at a higher risk of residual or recurrent cervical lesions if treated using cryotherapy instead of LEEP^29,30^ and if tumor excision is incomplete,^31^ even though the WHO recommendations for follow-up among screen-positive WLHIV do not vary by treatment method.^32^

In our analysis, abnormal colposcopic findings were found on the cervices of 3.5% (9/258) of WLHIV, despite testing hr-HPV negative. The respective rates were 4.5% (6/132) in the rural group and 2.4% (3/126) in the urban group. These WLHIV were of interest, because if only hr-HPV DNA testing had been used to screen them, potentially precancerous lesions would have been missed, as they would not have been recalled for follow-up VIA or mobile colposcopy. With quality control lagging behind the adoption of hr-HPV testing platforms, in LMICs, false negative tests may result in significant lesions being missed. A second screening test that relies on visual inspection of the cervix will thus be crucial in such high-risk patients. To further buttress this point, in this group of hr-HPV-negative but visual inspection ‘positive’ WLHIV, histopathology following LEEP revealed CIN III lesions in 2 women, both of whom belonged to the urban group. Representative colposcopic images obtained before and after acetic acid application in these 2 women are shown in Supplemental Figures 2 and 3. In light of this, a concurrent approach when these women present at a facility, by way of hr-HPV DNA testing and visual inspection (as used in our study) may be more cost-effective than recalling hr-HPV-positive WLHIV for colposcopy at a later date. This will also reduce loss to follow-up.

Inadequate facilities and high patient loads have been highlighted as key barriers to cervical precancer screening integration in HIV care.^33,34^ Due to high patient volumes and limited clinic spaces in rural and urban HIV clinics alike, it may not be possible to integrate cervical screening immediately using a screen, triage, and treat approach solely using existing staff and infrastructure. One feasible approach may be a more gradual coordination between HIV clinics and district and regional hospitals via a coordinated referral and data system that enables resources and staff to be used in screening other high-risk cohorts.

Women screened by the CCPTC who wish to, and can afford it, are given the option to get further management at other institutions. This means there may be some screen positives who have been managed in other institutions that we still may consider as loss to follow up. A coordinated national screening program may address this. Significantly, 19 out of the 22 women screened (n = 13 and n = 6 from the rural and urban settings, respectively) had cervical lesions that were not treated. None of these lesions qualified for ablative treatment (cryotherapy/thermal coagulation) using the WHO criteria.^35^ All of them had type 3 transformation zone and/or lesions extending into the endocervical canal, upper limit not seen. Ablative treatment could result in residual precancer in the endocervical canal that could progress to cancer; thus, a ‘see and treat approach’ is difficult to implement in this population. Seven of these women who had major changes as cervical findings (dense acetowhitening) were counseled for a LEEP while 12 with minor changes (thin acetowhitening) were given the option of biopsy/treatment with LEEP or follow-up screening in 6 months/1 year as the lesion could regress. This shows the difficulty in screening in this population because if there was unlimited funding, an endocervical curettage for histopathology or a Pap smear would have been performed to exclude a high-grade lesion in the endocervical canal. WLHIV, especially in a largely unscreened population, are also likely to have large cervical lesions not amenable to treatment by ablation.^36,37^ These underscore the need for countries/communities with a high prevalence of HIV to invest in training health workers to perform excisional treatments like LEEP as training middle cadre staff to perform ablative treatment alone will not be enough to cater for this population.

### Strengths and Limitations

To the best of our knowledge, this is the first study to focus on rural-urban differences in cervical screening outcomes among WLHIV in Ghana utilizing concurrent hr-HPV DNA testing and mobile colposcopy by trained nurses. The findings described are expected to influence the promotion of a cervical precancer policy targeting WLHIV in Ghana. Our study, however, has some noteworthy limitations. First, because we did not originally screen the WLHIV in the context of a research study, we did not perform an a priori power calculation to determine an appropriate sample size; thus, it is unclear whether the study was sufficiently powered to show differences in the outcome measures. Second, we recruited the participants conveniently among women presenting for scheduled reviews at the HIV clinics of the screening sites. Thus, the study samples may not be representative of WLHIV in either setting. Third, we did not differentiate between transient and persistent infections among the WLHIV in our analysis. Again, we did not perform full HPV genotyping for hr-HPV-positive WLHIV, which would have allowed a detailed hierarchical analysis of genotype distribution between the two settings. Likewise, we did not follow-up WLHIV with normal colposcopy findings and so could not determine how many lesions would have been missed if standalone mobile colposcopy was used to screen this cohort of WLHIV. Last, we were unable to assess additional clinical variables related to HIV care apart from those reported by participants themselves, such as viral load and HIV type.

## Conclusion

While plans are underway to adopt an integrative screening program targeting WLHIV as a special population in Ghana, our research work shows a need to increase education and access to HPV vaccination among WLHIV in both rural and urban areas. The present study findings are limited by resource limitations and logistical challenges in following up WLHIV counseled for conservative management and with some pending treatment in both rural and urban settings, demonstrating the difficulty faced in reaching early diagnosis and treatment, regardless of their area of residence.

### Recommendation for Screening

WLHIV have a high prevalence of hr-HPV and an increased risk of cervical cancer. Concurrent HPV DNA testing with a visual inspection method (colposcopy/VIA) reduces loss to follow-up compared to performing HPV DNA testing as a standalone test and recalling hr-HPV positive women for follow up with a visual inspection method. Concurrent HPV DNA testing and a visual inspection method may also pick up precancerous cervical lesions that are hr-HPV negative and may be missed if HPV DNA testing is performed alone.

## Supplemental Material

Supplemental Material - HPV DNA Testing and Mobile Colposcopy for Cervical Precancer Screening in HIV Positive Women: A Comparison Between Two Settings in Ghana and Recommendation for Screening Supplemental Material for HPV DNA Testing and Mobile Colposcopy for Cervical Precancer Screening in HIV Positive Women: A Comparison Between Two Settings in Ghana and Recommendation for Screening by Kofi Effah, Richard Anthony, Ethel Tekpor, Joseph E. Amuah, Comfort M. Wormenor, Georgina Tay, Smith E. Y. Kraa, Angela M. Katso, Christiana A. Akonnor, Seyram Kemawor, Stephen Danyo, Bernard H. Atuguba, Nana O. Mensah Essel, and Patrick K. Akakpo in Cancer Control

## Data Availability

The data that support the findings of this study are available from the corresponding author upon reasonable request.

## Abbreviations

CCPTC: Cervical Cancer Prevention and Training Centre
CIN: Cervical intraepithelial neoplasia
CI: Confidence interval
EVA: Enhanced Visual Assessment
HAART: Highly-active antiretroviral therapy
hr-HPV: High-risk human papillomavirus
HIV: Human immunodeficiency virus
IQR: Interquartile range
LEEP: Loop electrosurgical excision procedure
SD: Standard deviation
VIA: Visual inspection with acetic acid
WLHIV: Women living with HIV
