# Supplementary figures and images for: HPV DNA Testing and Mobile Colposcopy for Cervical Precancer Screening in HIV Positive Women: A Comparison Between Two Settings in Ghana and Recommendation for Screening
Source: Cancer Control. 2024 Apr 2;31:10732748241244678. doi: 10.1177/10732748241244678 (PMC10989037; doi:10.1177/10732748241244678)

Supplemental figure 1

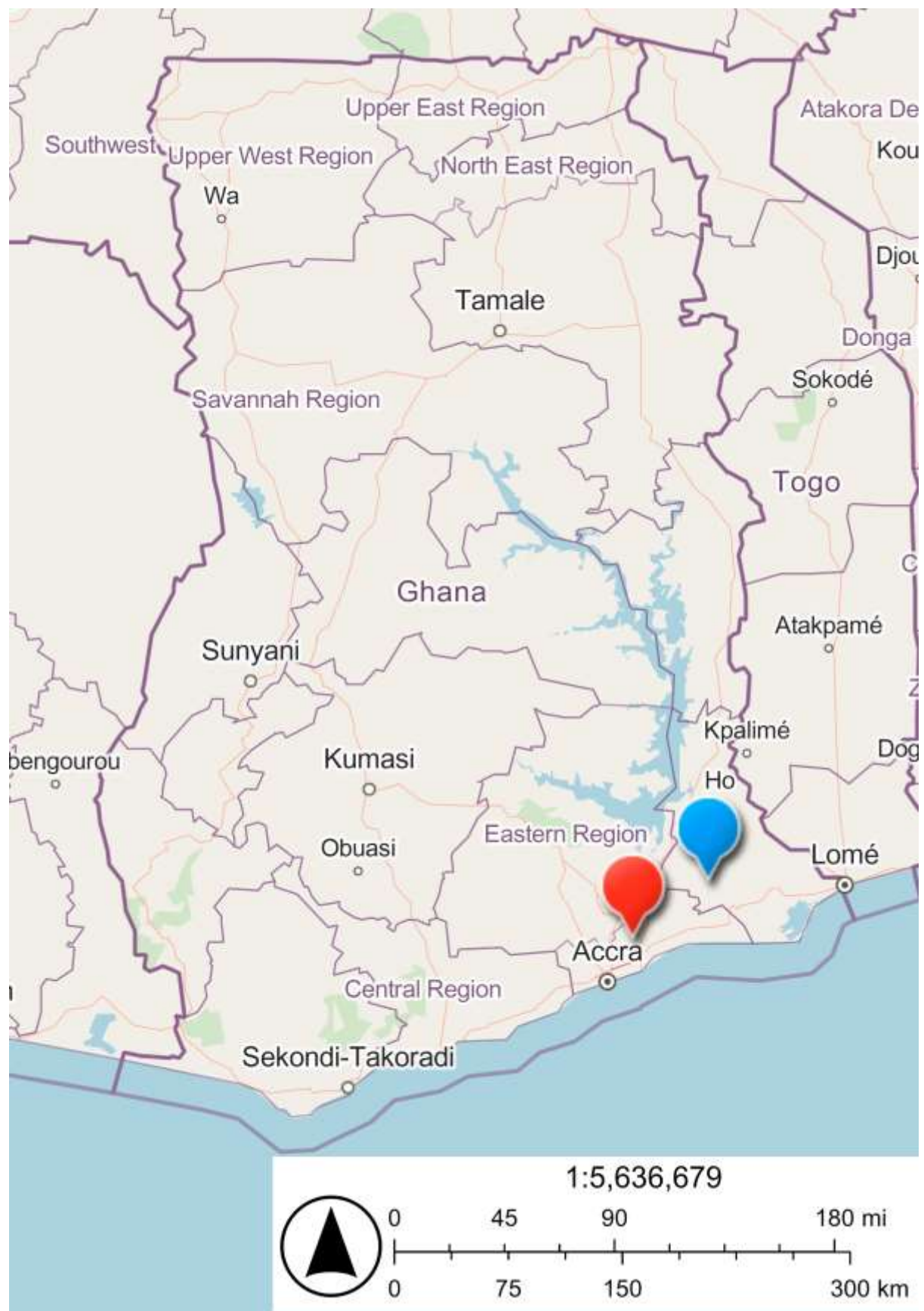

Supplemental figure 2

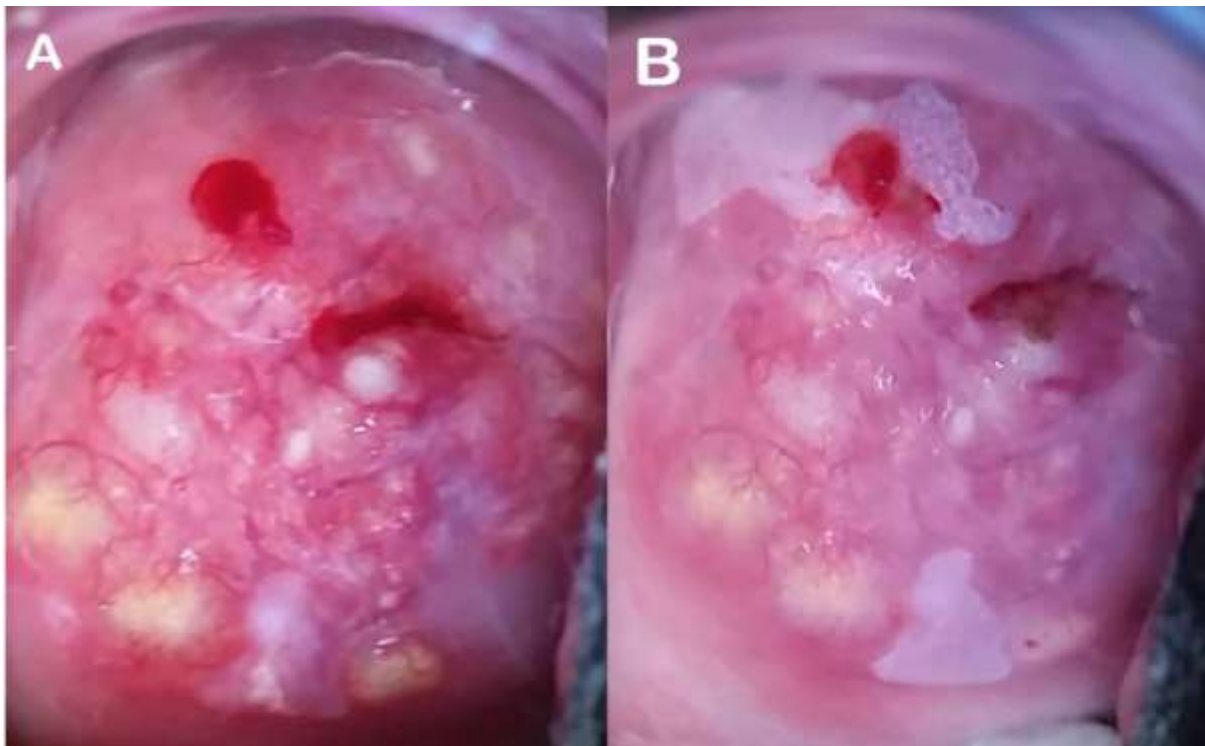

Supplemental figure 3

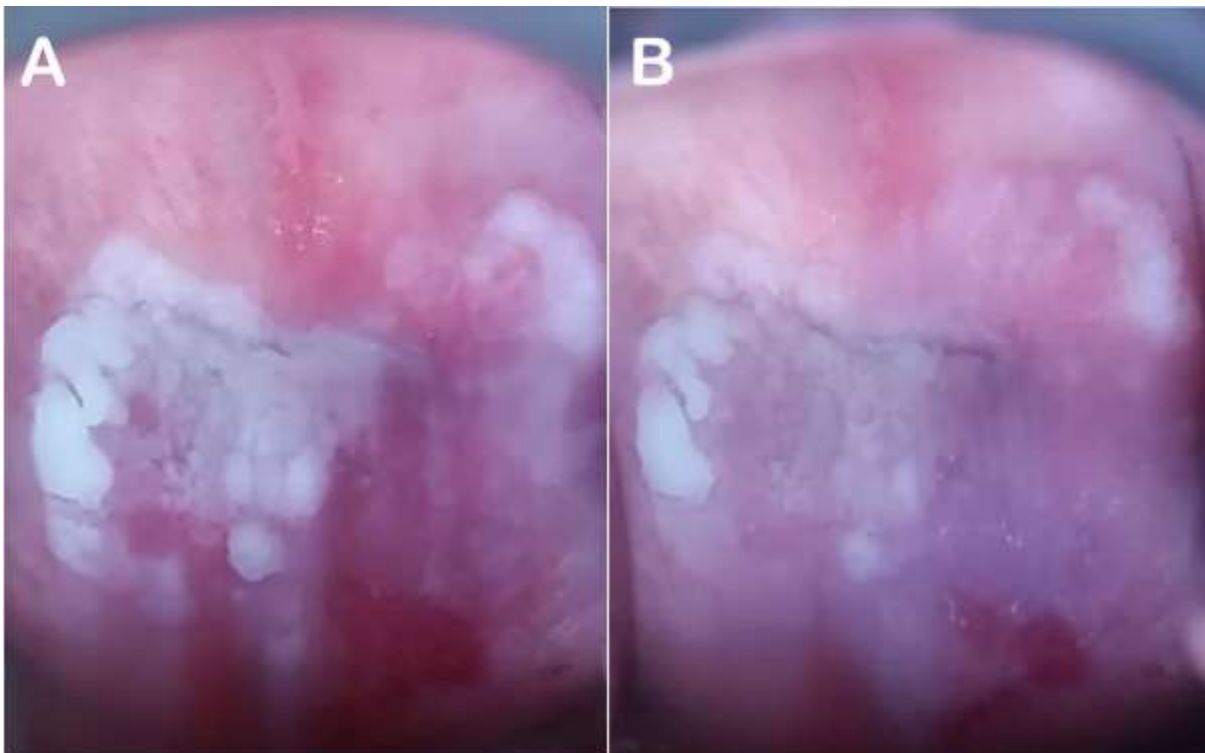

Supplement: Supplemental Material - HPV DNA Testing and Mobile Colposcopy for Cervical Precancer Screening in HIV Positive Women: A Comparison Between Two Settings in Ghana and Recommendation for Screening [file sj-pdf-1-ccx-10.1177_10732748241244678.pdf]
